# Quality Characteristics and Storage Stability of Guava Nectar Formulated with Natural Sweeteners

**DOI:** 10.17113/ftb.63.04.25.8807

**Published:** 2025-12-26

**Authors:** Muskaan Gupta, Swati Kapoor, Manju Bala, Bal Vipin Chandra Mahajan

**Affiliations:** 1 Department of Food Science and Technology, Punjab Agricultural University, Ludhiana, 141004 Punjab, India; 2 ICAR - Central Institute of Post-Harvest Engineering and Technology, Ludhiana, 141004 Punjab, India; 3 Punjab Horticultural Postharvest Technology Centre, Punjab Agricultural University, Ludhiana, 141004 Punjab, India

**Keywords:** bioactive compounds, rheological behaviour, *in-vitro* bioavailable iron, 5-hydroxymethyl furfural, non-enzymatic browning

## Abstract

**Research background:**

Recently, extensive use of refined sugars and artificial sweeteners has led to negative health implications. Therefore, this study explores natural or unrefined sweeteners such as honey, date syrup and jaggery as potential alternatives due to their nutritional and therapeutic properties.

**Experimental approach:**

The study aims to optimize the amounts of honey, jaggery and date syrup to substitute the addition of sucrose for sweetness in guava nectar prepared using two processing treatments: hot filling and cold filling. The bioactive and rheological properties, mineral composition (*in-vitro* bioavailable iron) and storage stability of the nectar were further evaluated. During storage, the formation of 5-hydroxymethylfurfural (HMF), effects on antioxidant activity and non-enzymatic browning were monitored to assess changes in overall quality.

**Results and conclusions:**

The amount of sucrose substitution in guava nectar was optimized at mass fractions of 50, 25 and 30 % for honey, jaggery and date syrup, respectively, based on organoleptic properties. The optimized formulations showed a significant improvement in total phenolic content and radical scavenging activity. The guava nectar showed pseudo-plastic behaviour with a weak gel structure due to the dispersion of pulp particles, which contributed to its viscoelastic nature at low strain (<10 %). The substitution of sucrose with natural sweetener resulted in increased mineral content; however, the bioavailability of iron considerably decreased. During storage, degradation of ascorbic acid and colour, acceleration of non-enzymatic browning and development of 5-hydroxymethyl furfural were notably high by the end of the sixth month, but the formulations remained microbiologically stable.

**Novelty and scientific contribution:**

New products can be formulated using natural sweeteners instead of sucrose, which may offer higher nutritional and therapeutic value. However, in this study, the product could be improved by further research to reduce negative effects on quality characteristics during storage.

## INTRODUCTION

Fruit-based beverages are widely consumed and form a significant part of urban households. Due to a paradigm shift in consumer preference towards healthier options over carbonated and artificially flavoured soft drinks, a massive surge in the fruit juice beverage market has been observed globally. Natural fruit juices are mainly composed of glucose and fructose, while commercially available ready-to-serve or nectars contain appreciable amounts of refined sugars in the form of sucrose or high fructose corn syrup (HFCS), which are deliberately added to increase sweetness. Excessive consumption of ready-to-serve beverages poses negative health implications as they are characterised by a high glycaemic index, causing rapid rise in blood glucose and insulin levels, along with increased levels of reactive oxygen species, inflammatory mediators and triglycerides in the human body, subsequently increasing the risk of diabetes mellitus and cardiovascular diseases. Moreover, the low satiety value of refined sugars leads to overconsumption of beverages, resulting in obesity (*1*, *2*).

To address the health risks associated with fruit beverage consumption, unrefined and artificial sweeteners are recognised as potential alternatives to refined sugars. Artificial, non-nutritive and low-calorie sweeteners, including sugar alcohols, can be used in fruit beverages to reduce calorie intake and help prevent obesity. However, studies have identified artificial sweeteners as contributing factors to various health issues such as coronary heart disease, stroke and mortality (*3*). A recent report by the World Health Organization (WHO) has also found that excessive consumption of non-sugar sweeteners can significantly increase the risk of type 2 diabetes, cardiovascular diseases and mortality (*4*). Steviol diterpene glycosides (150-450 times sweeter than sugar) are also widely used as natural sweeteners but may have side effects such as mutagenicity, reduced fertility and allergenic reactions (*5*). Moreover, stevia leaf extracts may not provide the desired consistency and mouthfeel in a beverage compared to sugar. Overall, it is important to continue exploring alternatives to refined and artificial sweeteners.

Thus, unrefined natural sweeteners such as honey, jaggery and date syrup could be explored in beverage production, as they not only contain significant nutritional compounds such as vitamins and minerals, but also have abundant health promoting properties due to the presence of organic acids, minerals and polyphenolic compounds (*5*-*7*). Previous studies have investigated the use of honey, jaggery and date syrup as substitutes for sucrose in beverages, dairy products and baked goods, revealing significant changes in the bioactive profile, rheological properties and colour aspects (non-enzymic browning) of the final products (*8*-*10*).

Guava is known to be effective in treating diarrhoea, hypertension, eczema, pain, dental caries, toothache and in boosting immunity. However, its consumption should be restricted in pregnant and lactating women (*11*). Red-fleshed guava, being most suitable for processing, is a widely consumed fruit in the beverage industry. It is also a rich source of citric, ascorbic, malic and succinic acids (*12*), as well as flavones, flavonols, flavonones and polyphenolic compounds such as gallic, chlorogenic, caffeic, *trans*-cinnamic, vanillic, *p*-coumaric, syringic, ferulic and ellagic acids, and is particularly rich in carotenoids such as all-*trans*-lutein, zeaxanthin, β-cryptoxanthin, α-carotene and β-carotene (*13*, *14*).

Hence, this investigation aims to explore the use of honey, jaggery and date syrup as potential alternatives to sucrose in guava nectar and to study the developed product for its impact on qualitative characteristics, including bioactive compounds, rheological behaviour, mineral composition and *in vitro* iron bioavailability. Furthermore, a six-month storage study was conducted to analyse the effect of substitution on various quality parameters such as colour, non-enzymatic browning and the development of 5-hydroxymethylfurfural.

## MATERIALS AND METHODS

### Procurement of raw materials and chemicals

Ripe red-fleshed guava (*Psidium guajava* var. Punjab Pink) was procured from Punjab Organic Vegetable and Fruit Producer Co. Ltd., Patiala, Punjab. Honey (Markfed Sohna^TM^, Jalandhar, India), date syrup (Lion^TM^, Tamil Nadu, India), cane jaggery powder (Vedaka^TM^, Nawanshahr, India) and sucrose (good quality refined crystal sugar) were obtained from the local market in Ludhiana, India. Chemical reagents (AR grade) were purchased from Sisco Research Laboratories Pvt. Ltd., Mumbai, India.

### Preparation of guava nectar

Different formulations of guava nectar in which addition of sucrose was substituted with unrefined natural sweeteners were prepared by following a standard method described by Sidappa *et al.* (*15*), where total soluble solids (TSS) and acidity of the guava nectar were maintained at minimum 15 °Bx and 0.3 % (maximum 1.5 %), respectively, according to standard specifications laid by FSSAI (*16*). The addition of sucrose was substituted with honey and jaggery at mass fractions of 25, 50, 75 and 100 % of, while date syrup substitution was 20, 30, 40 and 50 %, based on preliminary trials. Control was prepared using 100 % sucrose. The formulations were designed on the basis of TSS and acidity of raw materials, *i.e.* guava pulp (TSS=10.2 °Bx and acidity=0.65 %), honey (TSS=82.2 °Bx and acidity=0.15 %), jaggery (TSS=97.8 °Bx and acidity=0.29 %) and date syrup (TSS=72.1 °Bx and acidity=0.47 %).

To prepare guava nectar (control (with added sucrose), or with the addition of honey, jaggery or date syrup), ingredients were weighed depending on the formulation. Cold syrup containing water, citric acid and sweeteners was prepared and filtered through a muslin cloth. Guava pulp was added to the syrup, and the nectar was homogenized. This was followed by two different processing treatments: cold filling and hot filling. In the cold-filling process, nectar was filled in pre-cleaned glass bottles without pasteurisation, while in the hot filling process, the nectar was pasteurised (at 82-85 °C for 1–2 min) and filled in glass bottles. The bottles were corked, sterilised in boiling water (100 °C) for 20 min, labelled and stored under ambient conditions (18–36 °C) for 6 months.

### Physicochemical properties

Total soluble solids were estimated using a handheld refractometer (Erma, Tokyo, Japan) with a scale ranging from 0 to 32 °Bx and were corrected to 20 °C (*17*). The pH was measured using a pH meter (S220; Mettler Toledo, Greifensee, Switzerland) calibrated with standard buffer solutions at values of 4.01, 7.00 and 9.21. Titratable acidity was estimated according to AOAC method 935.57 (*17*). Briefly, 10 mL of nectar sample (*V*_s_) were diluted to a final volume of 100 mL (*V*_f_), and 20 mL of aliquot (*V*_a_) were drawn and titrated with 0.1 M NaOH solution using 1 % phenolphthalein solution as an indicator. The appearance of a light pink colour was noted as the end point:


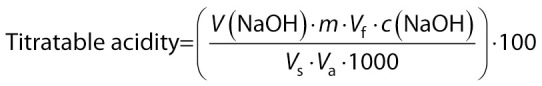
 /1/

where *m* is the equivalent mass of citric acid.

Lane and Eynon method was used for the estimation of reducing sugars (*18*). A mass of 4 g of nectar was diluted to 10–15 mL with distilled water and neutralised with 1 M NaOH using phenolphthalein indicator. A volume of 2 mL of 45 % lead acetate solution was added, the solution was kept for 10 min, and then precipitated with 5 mL of 22 % potassium oxalate solution. Final volume was made up to 100 mL. The solution was then filtered through Whatman filter paper no. 40. A volume of 5 mL of Fehling solution A and Fehling solution B was taken in a conical flask and boiled with simultaneous addition of 3 drops of 1 % methylene blue indicator. This was titrated against the sugar solution obtained within 1 min. Brick red precipitates were observed as the end point:


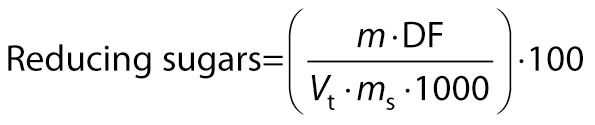
 /2/

where *m* is the mass of inverted sugar (mg), DF is the dilution factor, *V*ₜ is the titrant volume, and *m*_s_ is the mass of the sample.

Non-enzymatic browning was measured as absorbance at 440 nm (*A*_440 nm_) (*18*). A volume of 5 mL of nectar sample was diluted to 50 mL and centrifuged (3K30; Sigma Laborzentrifugen GmbH, Osterode am Harz, Germany) at 1000×*g* for 15 min at 4 °C. A volume of 2 mL of supernatant and 3 mL of alcohol were mixed thoroughly in a test tube. Absorbance was measured at 440 nm using aqueous alcohol (60 %) as a blank.

### Colour parameters

The L*, a* and b* values of the product were observed using CM-5 colour difference meter (Konica Minolta, Osaka, Japan). Chroma and hue were calculated as follows:


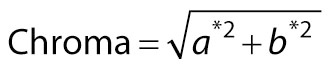
 /3/


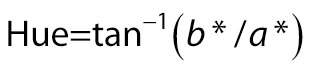
 /4/

### Organoleptic evaluation

To estimate consumer acceptability of the product, sensory evaluation was carried out using a 9-point hedonic scale for the following parameters: colour/appearance, mouthfeel, odour, flavour and overall acceptability, rated by 15 personnel including students and staff volunteers from the Department of Food Science and Technology, Punjab Agricultural University, Ludhiana, Punjab, India, who have basic understanding of the aforementioned terms. The personnel were briefed about the testing procedure on-site. Data on the personnel and demographic information were not collected as the primary objective of the study was to test the perceivable difference in the overall acceptability of the product following changes in the mass fractions of natural sweeteners rather than to correlate sensory preference with demographic variables (*19*).

### Bioactive compounds

Ascorbic acid (mg/100 mL) was estimated using titrimetric method as described by Bal *et al*. (*20*). A 2,6-dichloroindophenol dye (0.04 %) solution was standardized against the mixture of 5 mL of l-ascorbic acid solution (0.1 mg/mL of 0.4 % oxalic acid) and 5 mL of 0.4 % oxalic acid solution. The obtained volume of the titrant (*V*_t_) was used to calculate dye factor (DF) as follows:


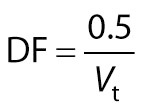
 /5/

A volume of 5 mL of sample (*V*_s_) was diluted to make up 50 mL with 0.4 % oxalic acid solution (*V*_f_). The solution was filtered and an aliquot of 20 mL (*V*_a_) was titrated with standardized 2,6-dichloroindophenol dye solution. Light pink colour persisting for at least 15 s was considered as the end point:


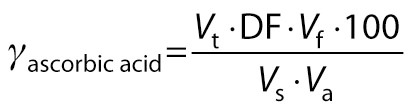
 /6/

where *γ*_ascorbic acid_ is the mass concentration of ascorbic acid (mg/100 mL), and *V*ₜ is the volume of titrant added to reach the endpoint of reaction

Total carotenoid and lycopene contents were determined in acetone-petroleum ether extract by plotting the absorbance (*A*) at 452 nm (using β-carotene standard curve) and 503 nm, respectively, as described by Lakhanpal and Vaidya (*8*):


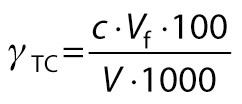
 /7/


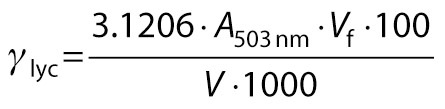
 /8/

where *γ*_TC_ is the mass concentration of total carotenoids (mg/100 mL), *γ*_lyc_ is the mass concentration of lycopene (mg/100 mL), *c* is the concentration of the respective standard solution, *V*_f_ is the final volume, *V* is the volume of the sample, and *A*_503 nm_ is the absorbance at 503 nm.

A volume of 5 mL of sample was ground with pestle in a mortar using acetone and anhydrous sodium sulphate until the residue turned colourless and formed a resinous mass. The liquid fraction was then transferred to a separating funnel, and 10–15 mL of petroleum ether were added to it. The pigments were extracted to petroleum ether by diluting acetone with water. The petroleum ether extract was filtered and the volume was made up to 25 mL. Absorbance was measured at 452 and 503 nm using a spectrophotometer (UV Vis 3500; Agilent Technologies, Petaling Jaya, Malaysia) and petroleum ether as a blank.

Total phenolic content (TPC) was determined using a method described by Swain and Hillis (*21*) with gallic acid as the standard. Methanolic extract (100 mL) of the sample was prepared by refluxing 5 mL of the sample with 80 % methanol for 2 h. Methanolic extract (0.2 mL) and 0.8 mL of distilled water were added to a test tube, followed by the addition of 5 mL of Folin-Ciocalteu reagent and 4 mL of saturated sodium carbonate solution. The formed solution was incubated for 45 min, the absorbance of the developed colour was measured at 765 nm (UV Vis 3500; Agilent Technologies), and the results were expressed as gallic acid equivalents in mg/100 mL.


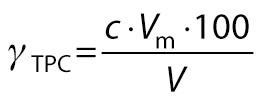
 /9/

where *γ*_TPC_ is the mass concentration of total phenolic content, *c* is the concentration of the standard solution, *V*_f_ is the final volume, and *V* is the volume of the sample.

DPPH radical scavenging activity was estimated as per Shimada *et al.* (*22*). Methanolic extract of the sample was prepared as described for total phenolic content. Methanolic extract (0.5 mL) was mixed with 0.5 mL of Tris buffer solution and 1 mL of 0.1 mM diphenylpicrylhydrazyl (DPPH) dye. Percentage of radical scavenging activity was compared to the control, which was prepared by mixing 0.5 mL of distilled water, 0.5 mL of Tris buffer and 1 mL of 0.1 mM DPPH. The solutions were incubated for 30 min, and the absorbance was measured at 517 nm (UV Vis 3500; Agilent Technologies). Results were expressed as radical scavenging activity (RSA/%) using the following formula:


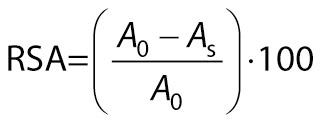
 /10/

where *A*_0_ is the absorbance of blank at 0 min and *A*_s_ is the absorbance of the sample after 30 min.

The 5-hydroxymethyl furfural (mg/100 mL) was measured as per modified Seliwanoff method (*8*). A volume of 20 mL of sample was diluted with water to 100 mL and centrifuged (3K30; Sigma Laborzentrifugen GmbH) at 5000x*g* for 15 min. The supernatant was filtered through Whatman no. 2 paper. Three successive extractions of 10-mL filtrate were done with 20 mL of ether in a separatory funnel after the addition of 2.5 g of NaCl. A volume of 1 mL of water was added to the obtained extract and evaporated at room temperature in an air draft. The volume of the residue was made up to 10 mL. A volume of 3 mL of extract was taken in a test tube with the addition of 3 mL of 99.9 % ethyl alcohol and 3 mL of 1 % resorcinol in HCl. The contents were mixed thoroughly and incubated in the dark for 30 min. The absorbance (*A*) was measured at 540 nm (UV Vis 3500; Agilent Technologies), and concentration was calculated using a standard curve of 5-hydroxymethylfurfural:


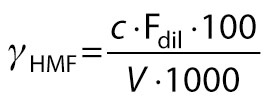
 /11/

where *γ*_HMF_ is the mass concentration of 5-HMF (mg/100 mL), *c* is the concentration of the standard solution, F_dil_ is the factor of dilution, and *V* is the volume of the sample.

### Rheological measurements

The rheological behaviour of nectar was analyzed using Physica MCR 101 rheometer (Anton Paar, Graz, Austria) equipped with concentric cylinder probe (DG 267/T 200/AL) having 25 mm inner diameter. Temperature was controlled precisely by the Peltier system.

#### Flow behaviour

Rheological flow behaviour was measured at shear rate 0–100 s^-1^ with 30 data points for each curve at 25 °C. The flow curve for shear stress (*τ*) *versus* shear rate was plotted and fitted to Ostwald-de-Waele and Herschel-Bulkley models in the following equations respectively using Rheoplus software:


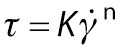
 /12/


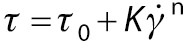
 /13/

where *τ*_0_ is the yield stress (Pa), is the shear rate (s^-1^), *K* is the consistency index (Pa·s) and *n* is the flow behaviour index.

#### Oscillatory sweeps

Amplitude sweeps were run to determine the impact of strain (0.01–100 %) on storage modulus (*G’*) and loss modulus (*G”*) at angular frequency 10 rad/s at 25 °C taking 25 data points. The data were recorded using Rheoplus software.

### Mineral composition and in vitro bioavailable iron

A volume of 5 mL of nectar was digested using 10 mL of concentrated nitric acid and concentrated perchloric acid in a ratio 3:1. The solution was kept overnight, followed by heating until a clear solution was obtained. The volume was made up to 25 mL and mineral content (Ca, K, Na, P, S, Mg, Mn, Cu, Zn, B and Fe) (reported in mg/L) was analyzed using inductively coupled plasma-optical emission spectrometry (5800 ICP-OES; Agilent Technologies).

Bioavailable iron was estimated using an *in vitro* method described by Rao and Prabhavati (*23*). A weighed amount of sample was digested using pepsin-hydrochloric acid (0.5 % pepsin in 0.1 M HCl) and incubated at 37 °C for 90 min after adjusting the pH to 1.35. The contents were centrifuged, and the filtrate was incubated again at 37 °C for 90 min after adjusting the pH to 7.5. The ionizable iron content of the extract was determined using atomic absorption spectrometry (Varian AA240FS; Agilent Technologies). *In vitro* bioavailable iron was calculated based on the prediction equation suggested by Rao and Prabhavati (*23*):


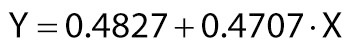
 /14/

where X is the percentage of ionizable iron at pH=7.5 and Y is the percentage of iron absorbed in adult men.

### Total plate count

To ensure the microbial load of the product remained within the prescribed limit of 50 CFU/mL as per FSSAI (*16*), the total plate count (CFU/mL) was determined using pour plate method with standard plate count agar medium (SRL, Mumbai, India) as per Ranganna (*18*).

### Statistical analysis

The data were statistically analysed using various ANOVA techniques with *post-hoc* Tukey’s test to evaluate significant differences between means, using STATISTIX v. 10.0. software (*24*).

## RESULTS AND DISCUSSION

### Effect of sucrose substitution with natural sweeteners and processing treatment on physicochemical and colour properties of nectar

The effect of sucrose substitution and processing treatment on the physicochemical properties and colour is shown in Table S1. The pH values of guava nectar with substituted sucrose increased with an increase in the mass fraction of honey, jaggery and date syrup. This trend was comparable to that reported by Cerevera-Chiner *et al.* (*10*), who observed an increase in pH from 3.58 to 3.75 in kiwifruit jam and from 3.45 to 3.82 strawberry jam as jaggery mass fraction increased from 0 to 75 %. The higher pH of jaggery may be due to the addition of lime during purification in jaggery production. Farahnaky *et al.* (*25*) reported the pH of date syrup of 4.24-4.62, and Belay *et al.* (*26*) reported that the pH of honey from different origins can range from 3.38 to 4.57. Therefore, it can be inferred that the higher pH of these sweeteners may have contributed to the increased pH of the guava nectar with substituted sucrose. The effects of hot filling and cold filling were found to be non-significant (p≤0.05).

An increase in reducing sugar content was observed with higher mass fractions of honey and date syrup, while the trend was reversed in jaggery-based guava nectar. The reducing sugar content decreased by up to twofold as the mass fraction of jaggery increased from 25 to 100 %. This may be attributed to the very high amounts of reducing sugars, namely glucose and fructose, in honey and date syrup (*26*-*28*). However, Cerevera-Chiner *et al.* (*10*) also reported a decrease in glucose and fructose content with increasing substitution with jaggery in strawberry and kiwifruit jams and suggested that sugars in jaggery may be less prone to hydrolysis during processing. Additionally, the higher pH of jaggery, due to residual lime from processing, may have interfered with the process of inversion. Reducing sugar contents were significantly higher (p≤0.05) in hot-filled than in cold-filled samples. Adulvitayakorn *et al.* (*29*) suggested that intensive heating leads to a breakdown of sucrose into glucose and fructose.

The amount of substitution of unrefined natural sweeteners also had a notable impact on the colour characteristics of red-fleshed guava nectar. It has been observed that, irrespective of the natural sweetener, L* values have significantly (p≤0.05) decreased, while a* and b* values increased with high mass fractions of substitution. Correspondingly, a change in chroma indicated enhanced saturation, and hue values indicated a loss of redness. An overall shift from redness to yellowness on the CIE colour wheel can be observed, representing a paradigm shift from characteristic pink colour to pink orange tonalities. This could be attributed to the presence of red, yellow and brown coloured compounds in natural sweeteners. The brown colour of unrefined sugars (jaggery) might be due to the presence of molasses, phytochemical pigments and amino acids. Additionally, the use of high temperatures during the processing of jaggery could also contribute to browning (*30*). However, the brown colour of honey depends on the composition of nectar, the process of acquisition, pigments present, temperature, light and storage time (*31*). Similarly, the colour of date syrup can vary from yellow to red-brown depending on the colour of the date flesh and processing temperature used to obtain the syrup, as explained by Julai *et al.* (*32*). They noted that the L* value of date syrup prepared by vacuum evaporation was twofold higher than of that obtained by open heating.

Although no apparent difference was visible to the naked eye between hot-filled and cold-filled samples at a given sweetener mass fraction, data in Table S1 show that heating has a pronounced effect on colour values. A statistically significant (p≤0.05) reduction in L* values and an increase in a* and b* values were observed in hot-filled samples compared to cold-filled ones. This may be due to the formation of Maillard reaction products during heating, which are brown in colour, as Tamanna and Mahmood (*33*) suggested that processing temperature may contribute to the formation of furoylmethyl derivatives in processed fruits and juices.

### Optimization of substitution based on organoleptic properties

Compared to the control guava nectar, mass fractions of 50 % honey, 25 % jaggery and 30 % date syrup were selected as sugar substitutes in hot-filled samples for further assessment (Table S2, Table S3 and Table S4), as panelists indicated that hot-filled samples had a richer mouthfeel than cold-filled samples. Increasing the substitution of sweeteners resulted in a darker product. In addition, the flavour profile was significantly affected at higher mass fractions. Honey imparted an astringent aftertaste, jaggery contributed caramelised notes and masked the guava flavour, while date syrup imparted an overly sweet aftertaste and a thick, gel-like consistency to the nectar. However, the results of the present organoleptic evaluation can be interpreted as a comparative assessment of guava nectar with substituted sucrose against control sample and cannot be considered as the marker of product acceptability among wider audience due to the lack of representative sample population of a particular demographic.

### Bioactive characterization of guava nectar with substituted sucrose

A significant change in the bioactive content of guava nectar with substituted sucrose compared to the control was observed (Table 1). Substitution of sucrose with natural sweeteners greatly improved the total phenolic content (TPC) and antioxidant activity. The highest values were found in date syrup-based guava nectar, followed by honey-based and jaggery-based guava nectar. Ascorbic acid content was also highest in date syrup-based guava nectar. However, the changes in honey-based and jaggery-based guava nectar were statistically non-significant (p≤0.05). These results support previous studies; Cerevera-Chiner *et al.* (*10*) also reported improved TPC and DPPH activity in jaggery-based strawberry and kiwifruit jams with increased substitution of sucrose.

**Table 1:** Bioactive compounds of guava nectar with different sweeteners

Bioactive compound	Control	Honey-based guava nectar	Jaggery-based guava nectar	Date syrup-based guava nectar
	*γ*(bioactive compound)/(mg/100 mL)			
Ascorbic acid	(14.8±0.2)^b^	(14.9±0.4)^b^	(14.5±0.4)^b^	(16.7±0.2)^a^
TPC (as GAE)	(85.3±0.2)^d^	(99.1±0.3)^b^	(94.4±0.3)^c^	(117.4±0.4)^a^
TC	(46.4±0.2)^a^	(41.6±0.1)^b^	(33.4±0.2)^d^	(34.1±0.1)^c^
Lycopene	(1.05±0.02)^a^	(0.96±0.03)^b^	(0.85±0.03)^c^	(0.94±0.03)^b^
	RSA/%			
	(56.4±0.2)^d^	(59.6±0.3)^b^	(58.5±0.4)^c^	(64.5±0.2)^a^

Data are presented as mean value±S.D. (*N*=3). Values with different letters in superscript in same row are statistically different (p≤0.05). TPC=total phenolic content, GAE=gallic acid equivalent, TC=total carotenoids, RSA=radical scavenging activity

The inherent presence of phenolic compounds in honey (gallic acid, chlorogenic acid, caffeic acid, coumaric acid, pinocembrin, chrysin, quercetin and abscisic acid), jaggery (gallic acid, protocatechuic acid, gentistic acid, 4-hydroxyphenylacetic acid, vanillic acid, syringic acid, *p*-coumaric acid and ferulic acid) and date syrup (catechin, caffeic acid, vanillic acid, syringic acid, ferulic acid, *p*-coumaric acid and sinapic acid) (*34*-*36*) may have contributed to the high phenolic content, which in turn improved the antioxidant potential of the product.

In contrast to the results reported by Lakhanpal and Vaidya (*8*), the substitution with natural, unrefined sweeteners resulted in a significant decrease in carotenoid and lycopene content in the beverage. It may be suggested that the presence of metal ions in honey, jaggery and date syrup (Table 1) contributed to the greater degradation of carotenoids during heat processing. Penicaud *et al.* (*37*) suggested that, particularly at low pH (which was found to be 3.44–3.97 in the prepared product), transition metals can oxidize carotenoids (unsaturated lipids). In addition, ascorbic acid content, which could act synergistically to prevent carotenoid oxidation, was not significantly higher in this case.

### Rheological characterization of guava nectar with substituted sucrose

The flow behaviour of guava nectar was studied using two different models: the Ostwald-de-Waele model and the Herschel-Bulkley model, as described in Table 2. Based on the obtained correlation coefficients, the Herschel-Bulkley model was a better fit for studying the characteristics of guava nectar. Table 2 presents rheological properties such as the flow behaviour index, consistency index and yield stress.

**Table 2:** Rheological parameters of Ostwald-de-Waele model and Herschel-Bulkley model in guava nectar with substituted sucrose

Natural sweetener	Ostwald-de-Waele model			Herschel-Bulkley model			
	*K*	*n*	R^2^	Yield stress	*K*	*n*	R^2^
Control	13.336	0.221	0.77	0.823	4.946	0.477	0.97
Honey-based guava nectar	8.749	0.372	0.92	10.937	2.188	0.629	0.93
Jaggery-based guava nectar	9.672	0.184	0.85	1.229	5.810	0.299	0.93
Date syrup-based guava nectar	17.548	0.205	0.83	1.253	9.789	0.349	0.94

*K*=consistency coefficient, *n*=flow behaviour index

**Fig. 1:**
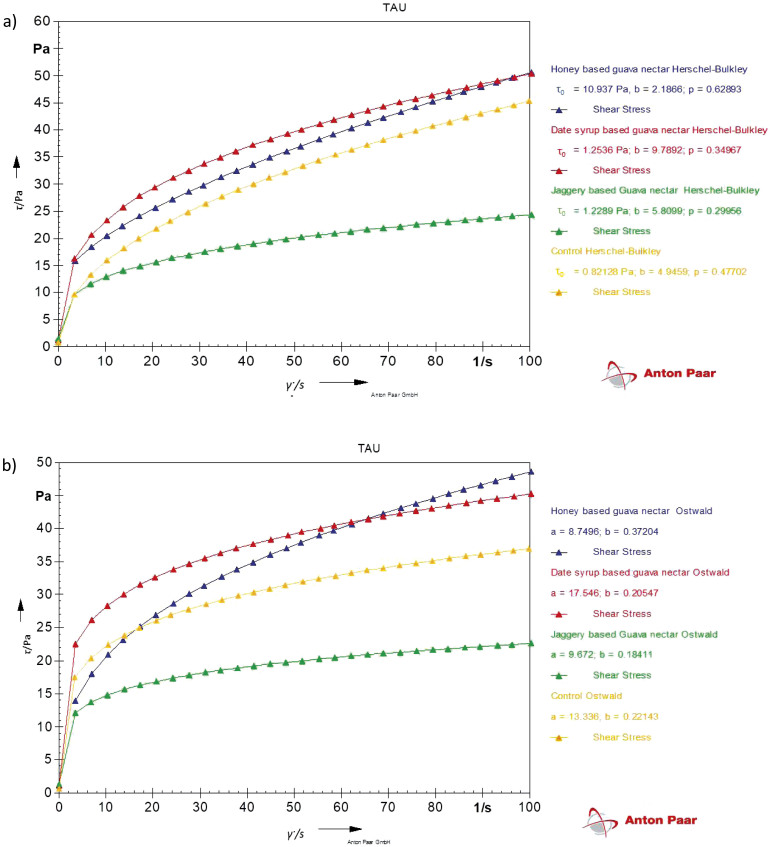
Steady state rheology for guava nectar with substituted sucrose fitted to: a) Herschel-Bulkley and b) Ostwald model

Herschel-Bulkley model (Fig. 1a) was found to have a better fit than Ostwald-de-Waele model (Fig. 1b). Flow curves fitted to the Herschel Bulkley model (Fig. 1a) showed an increase in shear stress with increasing shear rate. This indicated a decrease in viscosity with an increase in shear rate and values of the flow behaviour index for both the control and nectar with substituted sucrose were less than 1, representing shear-thinning behaviour. Therefore, guava nectar with substituted sucrose can be characterized as a Herschel-Bulkley fluid (*τ*_0_≠0). Similar results were reported by Peasura and Sinchaipanit (*38*), who substituted sucrose in guava nectar with neotame (0.01 %) and stevia (0.05 %), and observed shear-thinning behaviour.

However, the wide variation in rheological properties in control, honey-, jaggery- and date syrup-based guava nectar can be explained as an overall impact of high-temperature processing and variability in total solids, insoluble solids and pulp solids due to substitution with sweeteners, which can significantly affect viscosity. With higher amount of solids, the consistency coefficient increases and the flow behaviour index decreases (*39*). As suggested by Bhandari *et al.* (*40*), the viscosity of honey may be affected by the amount of monosaccharides and disaccharides, as the molecular chain length of sugars affects the viscosity of honey. The higher viscosity in date syrup-based guava nectar could be due to the presence of pectin and fibre. Furthermore, high temperatures during heating provide a higher molecular energy, which facilitates molecular movement and causes a decrease in the consistency coefficient. However, high-temperature processing can also alter the microstructure of the product and inactivate enzymes, causing a lesser degree of pectin degradation and leading to an increase in consistency (*39*). Therefore, a detailed study of sugar composition and its changes upon heating could be carried out to examine its effect on rheological parameters.

**Fig. 2:**
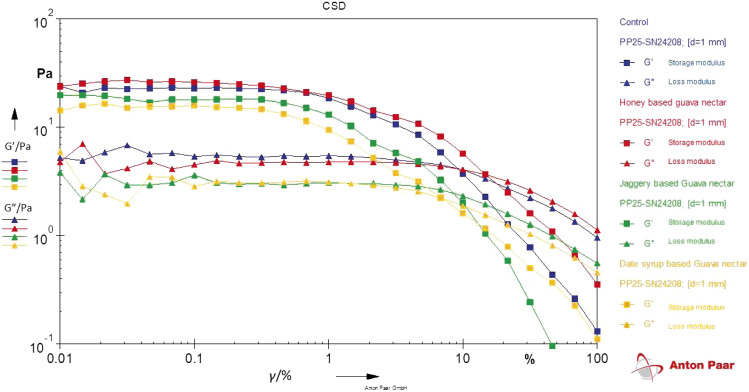
Amplitude sweeps for guava nectar with substituted sucrose. *γ*=shear strain

The graph of storage modulus (*G’*) and loss modulus (*G”*) plotted against strain (Fig. 2) shows that the value of *G’* was initially higher than *G”* for all samples, but *G’* became lower than *G”* when the strain increased. The graph shows that the solid structure was predominant. This could be due to the dispersion of pulp particles containing cell wall materials such as cellulose, hemicellulose, lignin and pectins in guava nectar, which may include fibre and represent a weak gel structure. The graph demonstrates that elastic properties were dominant over viscous properties, suggesting that the prepared nectars could be considered viscoelastic liquids under low strain amplitude (<10 %) and viscous liquids at higher strain amplitude. Similar results were reported by Augusto *et al.* (*41*) in peach juice with the addition of fibre (suspended solids) at 12.5 %, which showed higher *G’* than *G”* throughout. The study suggested that the addition of fibre caused a change from Newtonian behaviour to shear-thinning behaviour. Thus, it can be concluded that the prepared nectar exhibits a dispersion of insoluble polymeric clusters in a viscous medium composed of soluble polysaccharides, sugars and acids in water. Interaction between hemicellulosic and pectic polysaccharides forming a network could contribute to the elastic component of nectars (*41*).

### Mineral composition and *in vitro* bioavailable iron content of guava nectar with substituted sucrose

Mineral composition (Table 3) shows that substitution with honey, jaggery and date syrup at mass fractions of 50, 25 and 30 % respectively, significantly (p≤0.05) increased the contents of calcium, potassium, sodium, phosphorus, sulphur, magnesium, manganese and boron as honey, jaggery and date syrup are rich sources of minerals (*6*, *7*, *9*). Sugar substituted with date palm pulp meal in bread, and soursop drink with honey have also been reported to have higher mineral content by Obiegbuna *et al.* (*9*) and Olagunju and Sandewa (*42*), respectively. Iron was a focus in the present product, as guava is a rich source of organic acids and ascorbic acid, and studies suggest a synergistic effect of these compounds on the absorption of iron (*43*). Iron content was also found to be significantly (p≤0.05) higher in jaggery- and date syrup-based guava nectar and insignificantly in honey-based guava nectar than in the control. However, iron bioavailability was highest in the control (48.68 %), followed by honey-based (45.30 %), jaggery-based (30.86 %) and date syrup-based (27.25 %) guava nectar. This could be attributed to the lower acidity and higher pH (Table S1), as organic acids can have synergistic effects on enhancing iron absorption, as suggested by Teucher *et al.* (*43*). Govindaraj *et al.* (*44*) also concluded that the addition of citric acid and tartaric acid increased the bioavailability of iron in iron-fortified biscuits. Therefore, citric acid supplementation can be used to promote iron absorption in the body and in food products such as beverages, which are widely consumed and could serve as food vehicles to enhance iron absorption.

**Table 3:** *In vitro* bioavailable iron and mineral composition of guava nectar with sucrose substituted with natural sweeteners

Natural sweetener	*γ*/(mg/L)											
	Ca	K	Na	P	S	Mg	Mn	Cu	Zn	B	Fe	Fe*
Control	(86.5±0.3)^d^	(642.2±0.1)^d^	(45.2±0.2)^d^	(33.7±0.2)^d^	(183.7± 0.2)^d^	(42.7±0.3)^d^	(0.28±0.01)^c^	(0.20±0.01)^a^	(2.1±0.1)^bc^	(0.66±0.01)^d^	(3.28±0.06)^c^	(1.60±0.01)^a^
Honey	(99.2±0.2)^c^	(792.6±0.2)^c^	(53.6±0.1)^b^	(40.5±0.1)^c^	(201.93± 0.08)^c^	(49.1±0.2)^c^	(0.314±0.007)^c^	(0.21±0.01)^a^	(2.91±0.06)^a^	(1.16±0.02)^b^	(3.48±0.04)^bc^	(1.57±0.03)^a^
Jaggery	(117.2±0.1)^b^	(925.2±0.2)^b^	(148.3±0.1)^a^	(82.4±0.2)^a^	(229.5± 0.1)^a^	(80.3±0.2)^a^	(0.38±0.01)^b^	(0.257±0.007)^a^	(2.50±0.09)^b^	(0.94±0.01)^c^	(3.95±0.04)^a^	(1.22±0.01)^b^
Date syrup	(123.8±0.3)^a^	(1128.5±0.3)^a^	(46.073±0.06)^c^	(64.30±0.06)^b^	(221.7± 0.4)^b^	(67.4±0.2)^b^	(0.492±0.005)^a^	(0.22±0.01)^a^	(1.7±0.1)^c^	(1.37±0.01)^a^	(3.72±0.09)^ab^	(1.01±0.02)^c^

Data are represented as mean value±S.D. (*N*=3). Values with different letters in superscript in the same row are statistically different (p≤0.05). Mass fractions of sucrose substituted with natural sweeteners in guava nectar: honey 50 %, jaggery 25 % and date syrup 30 %. *Bioavailable iron

### Effect on qualitative characteristics during storage

During storage at ambient temperature for 6 months, extensive and crucial changes in qualitative characteristics were observed (Table 4). Reducing sugars increased during the first 4 months of storage, then decreased thereafter. Bal *et al.* (*19*) also reported an increase in reducing sugars in guava nectar during storage due to partial hydrolysis of starch by acid and the inversion of non-reducing sugars into reducing sugars. Moreover, a decrease in reducing sugar content during storage was observed, which could be explained by the involvement of reducing sugars in the formation of 5-HMF, found in the range from 6.6 to 12.5 mg/100 mL in the sixth month of storage. Cavaco *et al*. (*45*) also suggested that carbohydrate degradation during thermal processing may contribute to the formation of 5-HMF. The pH increased significantly (p≤0.05) over the period of six months, except in honey-based guava nectar. This could be due to the conversion of organic acids present in juices into simple sugars and salts due to the action of invertase (*46*).

**Table 4:** Effect on qualitative characteristics during storage

	*t*(storage)/month			
Quality parameter	0	2	4	6
	Control			
*w*(reducing sugar)/%	(5.1±0.1)^c^	(7.8±0.3)^b^	(10.8±1.6)^a^	(8.0±0.2)^b^
pH	(3.41±0.01)^b^	(3.43±0.01)^b^	(3.46±0.01)^a^	(3.46±0.01)^a^
*γ*_ascorbic acid_/(mg/100 mL)	(14.8±0.2)^a^	(10.8±0.4)^b^	(10.0±0.4)^bc^	(9.4±0.2)^c^
RSA/%	(56.4±0.2)^a^	(50.3±0.2)^b^	(26.6±0.4)^d^	(37.4±0.3)^c^
NEB	(0.052±0.001)^d^	(0.072±0.001)^c^	(0.087±0.002)^b^	(0.092±0.001)^a^
L*	(50.4±0.1)^a^	(48.8±0.2)^b^	(44.4±0.3)^c^	(44.7±0.3)^c^
a*	(15.4±0.2)^d^	(16.6±0.2)^c^	(20.2±0.2)^b^	(22.54±0.09)^a^
b*	(26.3±0.2)^d^	(28.3±0.1)^c^	(31.3±0.2)^b^	(31.9±0.1)^a^
*γ*_HMF_/(mg/100 mL)	BLD	BLD	(1.01±0.03)^b^	(5.89±0.03)^a^
	Honey-based guava nectar			
*w*(reducing sugar)/%	(10.5±0.3)^a^	(13.4±0.6)^a^	(13.4±0.4)^a^	(12.5±0.2)^a^
pH	(3.42±0.02)^a^	(3.44±0.01)^a^	(3.44±0.01)^a^	(3.45±0.02)^a^
*γ*_ascorbic acid_/(mg/100 mL)	(14.9±0.4)^a^	(9.6±0.4)^b^	(8.6±0.2)^c^	(7.5±0.4)^c^
RSA/%	(62.5±0.2)^a^	(43.5±0.4)^b^	(24.5±0.5)^d^	(36.5±0.3)^c^
NEB	(0.056±0.002)^d^	(0.069±0.001)^c^	(0.078±0.001)^b^	(0.095±0.001)^a^
L*	(47.3±0.3)^a^	(45.3±0.1)^b^	(42.6±0.2)^c^	(41.5±0.3)^d^
a*	(16.3±0.1)^d^	(17.21±0.08)^c^	(19.8±0.1)^b^	(20.1±0.1)^a^
b*	(34.3±0.1)^d^	(35.5±0.2)^c^	(39.82±0.07)^b^	(41.79±0.1)^a^
*γ*_HMF_/(mg/100 mL)	BLD	BLD	(0.93±0.03)^b^	(6.64±0.03)^a^
	Jaggery-based guava nectar			
*w*(reducing sugar)/%	(4.5±0.3)^c^	(5.1±0.1)^c^	(7.4±0.3)^a^	(6.6±0.3)^b^
pH	(3.81±0.01)^b^	(3.82±0.01)^b^	(3.84±0.01)^a^	(3.85±0.01)^a^
*γ*_ascorbic acid_/(mg/100 mL)	(14.5±0.4)^a^	(7.2±0.2)^b^	(6.5±0.2)^b^	(3.5±0.2)^c^
RSA/%	(58.5±0.4)^a^	(39.5±0.2)^b^	(24.6±0.4)^d^	(32.6±0.2)^c^
NEB	(0.069±0.001)^d^	(0.077±0.001)^c^	(0.089±0.003)^b^	(0.099±0.001)^a^
L*	(46.7±0.2)^a^	(45.3±0.1)^b^	(43.4±0.2)^c^	(37.3±0.2)^d^
a*	(17.6±0.2)^c^	(18.1±0.1)^b^	(18.5±0.2)^b^	(21.14±0.07)^a^
b*	(37.5±0.3)^d^	(40.4±0.2)^c^	(41.3±0.1)^b^	(44.67±0.09)^a^
*γ*_HMF_/(mg/100 mL)	BLD	BLD	(0.81±0.01)^b^	(7.74±0.06)^a^
	Date syrup-based guava nectar			
*w*(reducing sugar)/%	(7.9±0.1)^c^	(9.7±0.2)^b^	(12.4±0.4)^a^	(8.4±0.2)^c^
pH	(3.6±0.01)^b^	(3.67±0.01)^ab^	(3.68±0.02)^a^	(3.68±0.01)^a^
γ_ascorbic acid_/(mg/100 mL)	(16.74±0.2)^a^	(8.6±0.2)^b^	(7.7±0.2)^c^	(6.9±0.2)^d^
RSA/%	(64.5±0.2)^a^	(38.7±0.2)^b^	(27.3±0.3)^d^	(32.2±0.2)^c^
NEB	(0.105±0.001)^d^	(0.118±0.001)^c^	(0.172±0.002)^b^	(0.187±0.002)^a^
L*	(38.6±0.1)^a^	(37.3±0.3)^b^	(35.7±0.2)^c^	(34.4±0.3)^d^
a*	(21.4±0.2)^d^	(22.4±0.2)^c^	(23.2±0.2)^b^	(24.3±0.2)^a^
b*	(42.5±0.1)^b^	(47.3±0.2)^a^	(47.3±0.2)^a^	(47.5±0.2)^a^
γ_HMF_/(mg/100 mL)	BLD	BLD	(1.25±0.04)^b^	(8.44±0.04)^a^

Data are represented as mean value±S.D. (*N*=3). Values with different letters in superscript in the same row are statistically different (p≤0.05). NEB=non-enzymatic browning, HMF=5-hydroxymethylfurfural, RSA=radical scavenging activity, BLD=below limit of detection

Ascorbic acid degradation in guava nectar was prominent during storage, particularly in jaggery-based guava nectar. Ascorbic acid retention was lowest in jaggery-based guava nectar (23.81 %), followed by honey-based guava nectar (50 %), date syrup-based guava nectar (41.33 %) and the control (63.50 %). These observations were consistent with the results obtained by Hariharan and Mahendran (*47*), who reported a reduction in ascorbic acid during storage, as it is prone to oxidation and conversion into dehydroascorbic acid in ginger-lime ready-to-serve beverage sweetened with palmyra sugar. Therefore, the headspace in the glass bottle may have a considerable impact on the stability of ascorbic acid in the beverage. According to Tiwari *et al.* (*48*), ascorbic acid degrades aerobically at first and then anaerobically during storage in thermally processed orange juice. Sheraz *et al*. (*49*) suggested that the stability of ascorbic acid is also influenced by oxygen, temperature, pH of the medium, and the presence of metal ions such as Cu^2+^, Fe^2+^ and Zn^2+^, which catalyse degradation reactions. Therefore, it can be inferred that higher pH and the presence of metal ions (Table 3) in nectar with substituted sucrose may be responsible for reducing the stability of ascorbic acid in guava nectar.

Initially, DPPH radical scavenging activity decreased, then increased during the sixth month of storage. This could be attributed to the oxidation of phenolic compounds to their polymeric forms during storage (*50*). The results were in agreement with Klimczak *et al.* (*51*), who also reported a decrease in antioxidant activity during six months of storage in orange juices, followed by a sudden increase that is attributed to the formation of Maillard reaction products. This was evident in the present study, as 5-HMF content was significantly higher in the sixth month of storage.

Furthermore, an increase in non-enzymatic browning (NEB) values during storage, along with a decrease in L* values and subsequent increase in a* and b* values, indicates darkening of the product during storage. This could be attributed to chemical reactions such as oxidation of phenolic compounds and other reactions involving reducing sugars and organic acids, which can lead to the formation of brown pigments (*46*). Discolouration of juices due to the formation of brown pigments and the inherent dark colour of sweeteners is responsible for masking the characteristic pink colour of guava nectar and shifting its hue towards yellow.

By the end of storage, 5-HMF content was lower in the control than in the sugar-substituted guava nectar. Talcott *et al.* (*52*) also reported increased browning, higher amounts of 5-HMF and a decrease in L* values in passion fruit juice during 28 days of storage. It was also suggested that colour degradation is proportional to the loss of ascorbic acid, which is validated in the present study. Shinoda *et al.* (*53*) suggested that browning in orange juice, due to the formation of HMF and other browning compounds, is stimulated by the presence of ascorbic acid, sugars and citric acid, as well as by storage time and the absence of headspace, while the presence of chelating agents and radical scavengers inhibits the formation of compounds contributing to browning of juices. Additionally, the presence of metal ions such as Fe^2+^ and Cu^2+^ (as natural sweeteners are a rich source of minerals) promotes browning through Maillard reactions (*54*). Recent studies have raised concerns about the toxic potential, carcinogenicity and genotoxicity of 5-HMF. According to Abraham *et al.* (*55*), 5-HMF in the range of 80-100 mg/kg body mass per day can be consumed safely without adverse effects. Therefore, it can be concluded that guava nectar with substituted sucrose can be consumed safely up to the shelf life of six months. The total plate count increased significantly during the storage period of six months (0-5 CFU/mL), but remained below the limit specified by FSSAI (*16*), *i.e.* 50 CFU/mL. Microbiologically, guava nectar with substituted sucrose can also be considered safe for consumption for up to six months.

## CONCLUSIONS

Guava nectar with substituted sucrose can be successfully prepared using natural sweeteners: honey, jaggery and date syrup at substitution mass fractions of 50, 25 and 30 %, respectively, using the hot filling method. To maximise the health benefits of natural sweeteners, red-fleshed guava nectar with 30 % date syrup was found to have higher values of ascorbic acid content, total phenolic content and antioxidant activity than honey- or jaggery-based nectar. However, a considerable reduction in carotenoid and lycopene content was observed, which may be attributed to the presence of transition metals in the sweeteners. The substitution of natural sweeteners also led to a substantial improvement in the mineral content of the product, except for copper. Although iron content increased with the substitution of natural sweeteners, its bioavailability decreased, which could be associated with the higher pH than of the control, as the presence of organic acids has a synergistic effect on improving iron bioavailability. The study of rheological properties showed non-Newtonian (pseudoplastic) behaviour of the nectar due to the dispersion of pulp particles, which further contributed to the weak gel structure of the nectar, resulting in its viscoelastic properties below 10 % strain. Microbiologically, it could be safely stored at ambient temperature for six months. However, its degrading effects on quality parameters such as colour, non-enzymatic browning, ascorbic acid and 5-HMF development, which are highly correlated, must be considered, and methods for improvement could be suggested. Additionally, for further evaluation of market acceptance, sensory acceptance of the product should be tested among a wider audience with diverse participant pool among different demographics. Hence, based on the results, it can be concluded that the substitution with honey, jaggery and date syrup could be widely explored for the enrichment of the nutritional and therapeutic properties of beverages.

## Appendix

**Table S1:** Physicochemical and colour properties of guava nectar with sucrose substituted with different mass fractions of honey, jaggery and date syrup

*w*(natural sweetener)/%	Processing treatment	Parameter				
		pH	*w*(reducing sugar)/%	*L**	*a**	*b**
Control	HF	(3.39±0.01)^a^	(5.4±0.3)^a^	(50.3±0.1)^a^	(15.4±0.2)^a^	(26.7±0.2)^a^
	CF	(3.38±0.01)^a^	(4.5±0.2)^b^	(52.3±0.1)^b^	(13.7±0.4)^b^	(26.16±0.06)^b^
Honey 25	HF	(3.42±0.01)^cd^	(7.4±0.2)^e^	(51.17±0.04)^b^	(15.3±0.1)^e^	(29.36±0.08)^f^
	CF	(3.41±0.01)^d^	(6.5±0.3)^f^	(51.8±0.1)^a^	(13.4±0.2)^g^	(29.12±0.01)^f^
50	HF	(3.44±0.02)^bc^	(8.5±0.2)^d^	(47.3±0.3)^d^	(16.31±0.09)^d^	(34.3±0.1)^e^
	CF	(3.43±0.01)^cd^	(7.3±0.2)^e^	(48.4±0.2)^c^	(14.5±0.2)^f^	(34.4±0.3)^e^
75	HF	(3.48±0.01)^a^	(11.9±0.2)^b^	(45.93±0.05)^e^	(17.81±0.06)^c^	(38.1±0.1)^b^
	CF	(3.46±0.01)^ab^	(9.9±0.4)^c^	(46.3±0.1)^e^	(16.2±0.1)^d^	(36.7±0.1)^d^
100	HF	(3.49±0.01)^a^	(13.5±0.2)^a^	(43.47±0.08)^g^	(19.4±0.2)^a^	(38.6±0.2)^a^
	CF	(3.46±0.02)^ab^	(11.5±0.3)^b^	(44.71±0.05)^f^	(18.2±0.2)^b^	(37.5±0.1)^c^
Jaggery 25	HF	(3.72±0.01)^e^	(9.5±0.2)^a^	(46.7±0.2)^b^	(17.6±0.2)^d^	(37.5±0.2)^g^
	CF	(3.71±0.01)^e^	(9.3±0.3)^ab^	(48.2±0.2)^a^	(15.1±0.1)^g^	(40.8±0.2)^h^
50	HF	(3.97±0.01)^d^	(8.7±0.2)^bc^	(46.28±0.04)^c^	(18.54±0.09)^c^	(43.8±0.1)^e^
	CF	(3.96±0.02)^d^	(8.6±0.1)^c^	(47.8±0.1)^a^	(15.9±0.1)^f^	(41.3±0.1)^f^
75	HF	(4.22±0.01)^b^	(5.6±0.2)^d^	(44.4±0.2)^d^	(19.5±0.2)^b^	(49.2±0.1)^c^
	CF	(4.17±0.01)^c^	(4.8±0.2)^e^	(46.9±0.1)^b^	(16.4±0.1)^e^	(47.4±0.1)^d^
100	HF	(4.40±0.01)^a^	(4.30±0.06)^ef^	(38.6±0.2)^f^	(20.23±0.08)^a^	(52.4±0.2)^a^
	CF	(4.39±0.01)^a^	(3.8±0.1)^f^	(40.14±0.1)^e^	(17.6±0.1)^d^	(50.5±0.3)^b^
Date syrup 20	HF	(3.61±0.01)^de^	(6.14±0.08)^d^	(42.45±0.09)^b^	(20.9±0.1)^de^	(41.5±0.2)^g^
	CF	(3.59±0.01)^e^	(5.0±0.2)^e^	(46.6±0.1)^a^	(19.5±0.2)^f^	(41.31±0.07)^g^
30	HF	(3.65±0.01)^c^	(7.7±0.2)^b^	(38.6±0.1)^f^	(21.4±0.21^cd^	(42.5±0.1)^f^
	CF	(3.63±0.01)^cd^	(7.2±0.2)^bc^	(41.34±0.09)^c^	(20.6±0.2)^e^	(44.4±0.2)^e^
40	HF	(3.73±0.01)^b^	(8.6±0.2)^a^	(37.4±0.1)^g^	(23.3±0.3)^b^	(49.7±0.1)^c^
	CF	(3.73±0.01)^b^	(6.8±0.1)^c^	(40.89±0.09)^d^	(21.5±0.1)^c^	(48.7±0.2)^d^
50	HF	(4.28±0.01)^a^	(8.7±0.2)^a^	(35.8±0.1)^h^	(24.5±0.3)^a^	(52.6±0.1)^a^
	CF	(4.25±0.01)^a^	(7.5±0.3)^b^	(39.5±0.1)^e^	(21.8±0.2)^c^	(51.4±0.2)^b^

Data are represented as mean value±S.D. (*N*=3). Values with different letters in superscript in the same column (within each sweetener) are statistically different (p≤0.05). HF=hot-filled, CF=cold-filled

**Table S2:** Optimization of mass fraction of honey in guava nectar based on organoleptic evaluation

*w*(honey)/%	Processing treatment	Parameter				
		Colour	Odour	Mouthfeel	Flavour	Overall acceptability
Control	HF	(7.7±0.7)^a^	(7.5±0.7)^ab^	(7.600.6)^ab^	(7.7±0.3)^abc^	(7.6±0.5)^ab^
	CF	(7.8±0.6)^a^	(7.4±0.6)^abc^	(7.7±0.7)^ab^	(7.9±0.4)^ab^	(7.7±0.4)^ab^
25	HF	(7.7±0.6)^a^	(7.8±0.6)^ab^	(7.7±0.8)^ab^	(7.5±0.7)^abc^	(7.7±0.6)^ab^
	CF	(7.8±0.6)^a^	(7.9±0.4)^ab^	(7.8±0.4)^a^	7.7±0.4)^abc^	(7.8±0.4)^ab^
50	HF	(7.6±0.6)^a^	(8.3±0.3)^a^	(8.2±0.4)^a^	(8.2±0.4)^a^	(8.1±0.2)^a^
	CF	(7.7±0.6)^a^	(8.0±0.4)^ab^	(8.1±0.4)^a^	(8.1±0.4)^a^	(8.0±0.2)^a^
75	HF	(7.1±0.6)^ab^	(6.9±0.7)^bcd^	(6.9±0.6)^abc^	(6.9±0.6)^bcd^	(7.0±0.6)^abc^
	CF	(7.1±0.6)^ab^	(6.9±0.7)^bcd^	(6.7±0.8)^abc^	(6.7±0.8)^cd^	(6.9±0.7)^bc^
100	HF	(6.0±0.6)^b^	(6.0±0.7)^d^	(6.3±0.8)^bc^	(6.2±0.8)^d^	(6.1±0.7)^c^
	CF	(6.1±0.6)^b^	(6.1±0.7)^cd^	(6.0±0.6)^c^	(6.1±0.4)^d^	(6.1±0.6)^c^

Data are represented as mean value±S.D. (*N*=3). Values with different letters in superscript in the same column are statistically different (p≤0.05). HF=hot-filled, CF=cold-filled

**Table S3:** Optimization of different mass fractions of jaggery in guava nectar based on organoleptic evaluation

*w*(jaggery)/%	Processing treatment	Parameter				
		Colour	Odour	Mouthfeel	Flavour	Overall acceptability
Control	HF	(8.0±0.3)^ab^	(7.9±0.4)^abc^	(8.1±0.4)^ab^	(8.1±0.4)^ab^	(8.0±0.4)^abc^
	CF	(8.0±0.3)^ab^	(7.9±0.4)^abc^	(7.6±0.6)^abc^	(7.9±0.2)^abc^	(7.8±0.4)^abc^
25	HF	(8.2±0.3)^a^	(8.4±0.6)^a^	(8.5±0.6)^a^	(8.7±0.3)^a^	(8.4±0.4)^a^
	CF	(8.2±0.3)^a^	(8.1±0.4)^ab^	(8.0±0.8)^abc^	(8.4±0.6)^a^	(8.2±0.4)^ab^
50	HF	(7.8±0.3)^ab^	(7.7±0.6)^abcd^	(7.4±0.4)^abc^	(7.6±0.6)^abcd^	(7.6±0.4)^abcd^
	CF	(7.8±0.3)^ab^	(7.4±0.6)^abcd^	(7.3±0.4)^abc^	(7.4±0.6)^abcd^	(7.5±0.4)^abcd^
75	HF	(7.3±0.3)^bc^	(7.5±0.8)^abcd^	(7.0±0.8)^abc^	(6.7±1.0)^bcd^	(7.1±0.6)^bcd^
	CF	(7.2±0.3)^bc^	(7.0±1.0)^bcd^	(6.8±1.2)^bc^	(6.7±1.1)^bcd^	(6.9±0.8)^cd^
100	HF	(6.7±0.7)^c^	(6.7±0.7)^cd^	(6.4±1.0)^c^	(6.3±0.8)^d^	(6.5±0.7)^d^
	CF	(6.7±0.7)^c^	(6.0±0.7)^d^	(6.4±1.3)^c^	(6.4±1.0)^cd^	(6.5±0.8)^d^

Data are represented as mean value±S.D. (*N*=3). Values with different letters in superscript within the same column are statistically different (p≤0.05). HF=hot-filled, CF=cold-filled

**Table S4:** Optimization of different mass fractions of date syrup in guava nectar based on organoleptic evaluation

*w*(date syrup)/%	Processing treatment	Parameter				
		Colour	Odour	Mouthfeel	Flavour	Overall acceptability
Control	HF	(7.5±0.5)^a^	(7.9±0.2)^ab^	(7.8±0.4)^a^	(7.6±0.6)^a^	(7.7±0.2)^ab^
	CF	(7.6±0.6)^a^	(7.8±0.3)^abc^	(7.9±0.2)^a^	(7.5±0.5)^ab^	(7.7±0.3)^ab^
20	HF	(8.0±0.4)^a^	(8.1±0.4)^a^	(8.0±0.4)^a^	(7.9±0.6)^a^	(8.0±0.3)^a^
	CF	(7.9±0.2)^a^	(8.1±0.2)^a^	(8.2±0.6)^a^	(8.1±0.2)^a^	(8.1±0.1)^a^
30	HF	(7.9±0.4)^a^	(8.5±0.5)^a^	(8.3±0.4)^a^	(8.6±0.4)^a^	(8.3±0.4)^a^
	CF	(7.9±0.4)^a^	(8.3±0.4)^a^	(8.1±0.6)^a^	(8.4±0.2)^a^	(8.2±0.3)^a^
40	HF	(7.8±0.8)^a^	(7.7±0.8)^abc^	(7.6±0.6)^ab^	(7.6±0.6)^a^	(7.7±0.7)^ab^
	CF	(7.8±0.8)^a^	(7.5±0.5)^abc^	(7.5±0.7)^abc^	(7.5±0.5)^ab^	(7.6±0.6)^abc^
50	HF	(6.8±1.1)^a^	(6.6±1.3)^bc^	(6.3±1.0)^bc^	(6.3±1.0)^b^	(6.5±1.1)^bc^
	CF	(6.8±1.1)^a^	(6.4±1.0)^c^	(6.2±1.0)^c^	(6.3±1.0)^b^	(6.4±1.0)^c^

Data are represented as mean value±S.D. (*N*=3). Values with different letters in superscript within the same column are statistically different (p≤0.05). HF=hot-filled, CF=cold-filled
